# Effects of Maresin 1 (MaR1) on Colonic Inflammation and Gut Dysbiosis in Diet-Induced Obese Mice

**DOI:** 10.3390/microorganisms8081156

**Published:** 2020-07-30

**Authors:** Irene C. León, Sergio Quesada-Vázquez, Neira Sáinz, Elizabeth Guruceaga, Xavier Escoté, María Jesús Moreno-Aliaga

**Affiliations:** 1 Department of Nutrition, Food Science and Physiology, Centre for Nutrition Research, University of Navarra, 31008 Pamplona, Spain; ileon.5@alumni.unav.es (I.C.L.); nsainz@unav.es (N.S.); 2 Eurecat, Technology Centre of Catalunya, Nutrition and Health Unit, 43204 Reus, Spain; sergio.quesada@eurecat.org; 3 Bioinformatics Platform, Center for Applied Medical Research (CIMA) University of Navarra, 31008 Pamplona, Spain; eguruce@unav.es; 4 Navarra Institute for Health Research (IdiSNA), 31008 Pamplona, Spain; 5 Department of Biochemistry and Biotechnology, Universitat Rovira i Virgili, Campus Sescelades, 43007 Tarragona, Spain; 6 CIBERobn Physiopathology of Obesity and Nutrition, Carlos III Health Institute, 28029 Madrid, Spain

**Keywords:** MaR1, SPMs, dysbiosis, gut microbiota, colonic mucosa, inflammation

## Abstract

The aim of this study was to characterize the effects of Maresin 1 (MaR1), a DHA-derived pro-resolving lipid mediator, on obesity-related colonic inflammation and gut dysbiosis in diet-induced obese (DIO) mice. In colonic mucosa of DIO mice, the MaR1 treatment decreased the expression of inflammatory genes, such as *Tnf-α* and *Il-1β*. As expected, the DIO mice exhibited significant changes in gut microbiota composition at the phylum, genus, and species levels, with a trend to a higher Firmicutes/Bacteroidetes ratio. Deferribacteres and Synergistetes also increased in the DIO animals. In contrast, these animals exhibited a significant decrease in the content of Cyanobacteria and Actinobacteria. Treatment with MaR1 was not able to reverse the dysbiosis caused by obesity on the most abundant phyla. However, the MaR1 treatment increased the content of *P. xylanivorans*, which have been considered to be a promising probiotic with healthy effects on gut inflammation. Finally, a positive association was found between the Deferribacteres and *Il-1β* expression, suggesting that the increase in Deferribacteres observed in obesity could contribute to the overexpression of inflammatory cytokines in the colonic mucosa. In conclusion, MaR1 administration ameliorates the inflammatory state in the colonic mucosa and partially compensates changes on gut microbiota caused by obesity.

## 1. Introduction

Gut microbiota is a complex and dynamic entity which works as a metabolic organ, constituted by 10^9^ to 10^13^ bacteria, including approximately 500–1000 different bacteria species, which are mainly classified into two bacteria phyla: Firmicutes and Bacteroidetes [1]. Nowadays, it is known that gut microbiota plays an important role in the harvesting, storage, and expenditure of energy obtained from the diet, becoming a critical factor which contributes to the development of obesity [2,3]. Thus, microbiota contribute to the whole organism homeostasis. Despite the numerous beneficial aspects of the gut microbiota over host homeostasis, sometimes an excessive proliferation of particular species can be translated into an overproduction of some metabolites that can exert a harmful effect on the intestine and even cause a systemic inflammation in the worst scenario [4]. Therefore, a healthy host–microorganism balance is required in order to maintain proper immune and metabolic functions and prevent disease development [5]. In fact, preclinical studies using different mice models have shown that both obesity and high fat diet (HFD), containing a higher percentage of energy in the form of saturated lipids, led to gut microbiota dysbiosis characterized by an overgrowth of some bacteria phyla and a reduction of other phyla, which caused undesired consequences, such as intestinal inflammation or epithelial barrier disruption [6]. Dysbiosis is defined as an alteration or imbalance in the microbiota ecosystem, which generates a disruption of homeostasis state [7]. Several studies have shown important correlations between imbalances in the human intestinal microbiota composition and obesity and its associated diseases [2,3]. In animal models, the interaction between a HFD and gut microbiota acts as a potential source of proinflammatory molecules in the colon and the small intestine, an important risk factor in the progress of bowel inflammation [8,9]. Indeed, there is a consensus about the close link between inflammatory bowel disease and gut dysbiosis; however, a direct causal relationship between them has not been confirmed [10]. Obesity seems to aggravate the burden of inflammatory bowel disease in humans. However, the complex relationship between dysbiosis of gut microbiota and gut inflammation in bowel diseases still needs to be better elucidated in obese humans [11,12]. Dysbiosis is directly related to a higher intestinal permeability due to the epithelial barrier deterioration, increased lipopolysaccharide (LPS) levels in the circulation, small intestine bacterial overgrowth, tight junctions’ alteration, and even the whole bacterial translocation, among others, causing endotoxemia, which can reach and damage the liver through the portal vein [13]. A recent paper by Rosso et al. assessed, in a cohort of biopsy-proven NAFLD patients, the relation between Zonulin, an inactive precursor of haptoglobin-2 which plays a role in the regulation of the tight junctions, and a higher waist circumference, and suggested that visceral obesity generated a significant effect on the impairment of intestinal permeability [14]. Moreover, in some HFD models, it has also been suggested that gastrointestinal microbiome alterations can affect the pathogenesis of cardiometabolic diseases by enhancing its development through different pathways, including an increase of energy harvesting, a rise in metabolism harvesting, and a higher level of some proinflammatory cytokines expression [15]. It has been recognized that the proinflammatory cytokines are responsible for the inflammation of the intestinal mucosa, whose production and release is increased in related colon diseases, such as ulcerative colitis. These cytokines include tumor necrosis factor-α (TNF-α), interleukin-6 (IL-6), and interleukin-1β (IL-1β) [16].

Some studies have reported that consumption of marine origin omega-3 polyunsaturated fatty acids (n-3 PUFAs), such as eicosapentaenoic acid (EPA) and docosahexaenoic acid (DHA), contributed positively to the regulation of the systemic metabolism [17]. These molecules downregulated the production of proinflammatory cytokines and chemokines, reduced the amount of proinflammatory n-6 PUFA derivatives, and avoided the activation of the NF-κB pathway [17]. EPA and DHA are substrates for the constitution of endogenous specialized pro-resolving lipid mediators (SPMs), such as resolvins, protectins, and maresins, which have potent anti-inflammatory and pro-resolving capacities [18,19,20]. Consequently, beneficial actions of n-3 PUFAs on obesity-induced insulin resistance and inflammation have been partly related to the synthesis of SPMs [20,21]. Moreover, n-3 PUFAs-derived mediators such as 17(R)-hydroxy docosahexaenoic acid (17R-HDHA), aspirin-triggered resolvin D1 (RvD1), and resolving D2 (RvD2) have prevented inflammation in an experimental mice model of colitis [22].

Maresins (MaR) constitute a relatively novel family of DHA-derived SPMs [23]. Maresin 1 (MaR1, 7R,14S-dihydroxy-docosa-4Z,8E,10E,12Z,16Z,19Z- hexaenoic acid) biosynthesis is initiated in human macrophages from endogenous DHA [23,24]. MaR1 displays potent analgesic actions controlling local inflammation resolution and associated inflammatory pain [24]. In animal models of obesity, MaR1 attenuated HFD-induced WAT inflammation, reduced M1 macrophage markers, and increased the expression of adiponectin [21]. In adipocytes, MaR1 counteracted the alterations on lipolysis and autophagy machinery caused by proinflammatory cytokines [25]. Recent studies have also shown the effectiveness of MaR1 to regulate gastrointestinal tract functions. In this way, MaR1 has been shown to ameliorate liver steatosis in nonalcoholic fatty liver disease (NAFLD) animal models, inducing fatty acid oxidation genes and regulating autophagy and endoplasmic reticulum stress [26,27,28,29,30]. In mice with dextran sulfate sodium (DSS) or 2,4,6-trinitrobenzenesulfonic acid-induced colitis, MaR1 reduced leukocyte infiltration, inhibited neutrophil migration, and reduced the levels of the following proinflammatory cytokines: IL-1β, TNF-α, IL-6, and INF-γ. This protective effect of MaR1 in different models of experimental colitis seems to be mediated by the inhibition of NF-κB activity, the reduction of inflammatory mediators, and the promotion of the anti-inflammatory macrophage M2 phenotype [31]. Moreover, in rats with DSS-induced colitis, MaR1 also had a protective role through the activation of the Nrf2 signaling, leading to the inactivation of the TLR4/NF-κB signaling pathway, the reduction of proinflammatory mediators, and the regulation of intestinal tight junctions proteins [32]. Along these lines, a recent work by Castilla-Madrigal et al. has shown that some proinflammatory cytokines in the jejunal mucosa, whose gene expression was increased in diet-induced obese (DIO) mice, were decreased after treatment with MaR1 [33]. In this work, it has also been found that some of these SPMs (MaR1, RvD1, and RvD2) block TNF-α inhibition of intestinal sugar and glutamine uptake in Caco-2 cells [33].

Therefore, the aim of the current study is to investigate the potential ability of MaR1 to reverse the gut microbiota dysbiosis and to reduce the colonic inflammation in DIO mice.

## 2. Materials and Methods

### 2.1. Animal Models and Experimental Design

Twenty-three male C57BL/6J mice (seven weeks old) were obtained from Harlan Interfauna Ibérica (Spain). The mice were housed in plastic collective cages and kept under controlled environmental conditions at a temperature of 21 ± 2 °C, 12/12 h light/dark cycles, and humidity levels of 50 ± 10%. Experimental procedures were performed according to National and Institutional Guidelines for Animal Care and Use and after approval of the Ethics Committee for Animal Experimentation of the University of Navarra (approval ID: 047-15, May 15th 2015). Two different pelleted diets were used. The standard chow diet for the control group had a caloric profile contribution of 3.1 kcal/g (13% of kcal from fat, 67% from carbohydrates, and 20% from protein; Harlan Teklad Global Diets, Spain). The HFD which was used to induce obesity and insulin resistance in the C57BL/6J mice had a caloric profile contribution of 5.21 kcal/g (20% proteins, 20% carbohydrates, and 60% fat; Research Diets, New Brunswick, USA) [34]. The animals were fed with these diets *ad libitum* for 3 months [21]. After this period, the control group (*n* = 7) received a daily oral gavage of the vehicle (100 μL of saline solution with 0.1% ethanol) for 10 days. Additionally, the DIO mice were assigned to two subgroups that received a daily oral gavage of the vehicle (*n* = 8) or MaR1 (*n* = 8) (50 μg/kg body weight; Cayman, Ann Arbor, Michigan, USA) for 10 days [28] On the last day of treatment, fresh faeces were collected and immediately frozen at −80 °C for future DNA extraction and metagenomics analyses. Once the treatment was concluded, animals were sacrificed after 12 h of fasting. Colons were carefully collected and stored at −80 °C. The colonic mucosa was excised carefully by scraping the last colon’s section [35].

### 2.2. DNA Extraction Microbiome

Total faecal microbial DNA extractions were performed using the QIAampDNA Stool Mini Kit (Qiagen, Hilden, Germany), as previously described in other studies [36]. The DNA concentration was quantified using the spectrophotometer NanoDropND-1000 (Thermo Scientific, Waltham, MA, USA) and samples were storage at −80 °C until their use.

### 2.3. Metagenomic Analysis

The Next Generation Sequencing was performed in a MiSeq platform (Illumina, San Diego, CA, USA). For each DNA sample, the V3–V4 hypervariable regions of 16S rRNA gene were amplified using specific primers. These hypervariable regions were used to characterize many different bacteria in a sample, to quantify microbiota at the different phylogenetic levels, and also to discard genetic contaminations from mice bowel tissue [37]. A second PCR is needed in order to introduce the specific index for each sample and the Illumina adaptors (FC-131-1002, Illumina). The PCR clean-up was performed with AMPure XP and the amplicons were checked with a LabChip, (2100 Bioanalyzer, Agilent Technologies Spain). To conclude, all the libraries were pooled at 8 pM concentration in a single run of MiSeq sequencer (MS-102-2003 MiSeq^®^ Reagent Kit v2, 500 cycle) (Illumina) at the Bioinformatics and Genomics Services (University Autonomous of Barcelona, Spain). For the metagenomics studies, >100,000 reads per sample were procured to analyse the bacterial composition 16S rRNA sequences. The obtained sequences were filtered following quality criteria of LotuS processing protocol for OTUS (Operational Taxonomic Units) [38] (release 1.58). This pipeline includes UPARSE de novo sequence clustering [39], removal of chimeric sequences, and phix contaminants [40] for the identification of OTUs and OTU abundance matrix generation. Finally, taxonomy was assigned using BLAST [41] and HITdb [42] achieving up to species-level sensitivity. The abundance matrices were first filtered, and then normalized in R/Bioconductor [43] at each of the following classification levels: OTU, species, genus, family, order, class, and phylum. Briefly, taxa were discarded for future analysis when less than 4 reads were obtained, which occurred in more than 50% of the samples of all the experimental conditions, and a global normalization was performed using the library size as a correcting factor and log2 data transformation.

### 2.4. Gene Expression in Colonic Mucosa by qRT-PCR

Total mRNA extraction from the colonic mucosa was obtained using TRIzol and mechanical collision making use of homogenizer Ultra-Turrax T25 basic (IKA-Werke, Staufen, Germany). The RNA was resuspended in RNAsa free water (Ambion, Austin, TX, USA), quantified with a NanoDropND-1000, treated with a DNase (Ambion, Austin, TX, USA) to remove residual DNA, and retrotranscribed to cDNA with M-MLV (Invitrogen, Carlsbad, CA, USA). Gene expression was analyzed in a real-time PCR system 7900HT (Applied Biosystem, Foster City, CA, USA), using TaqMan gene expression assays for *Il-1β*, *Il-6*, *Mcp-1*, and *Tnf-α* and normalized to the 18S gene (Table S1). Gene expression for leucine-rich repeat domain-containing G protein-coupled receptor 6 (*Lgr6*) was also analyzed and normalized to the 36B4 gene by Power SYBR Green PCR (Bio-Rad, München, Germany). Primers were as follows: *Lgr6* Fw ATCATGCTGTCCGCTGACTG, *Lgr6* Rv ACTGAGGTCTAGGTAAGCCGT, *36b4* Fw CACTGGTCTAGGACCCGAGAAG, and *36b4* Rv GGTGCCTCTGGAGATTTTCG. The relative changes in gene expression were calculated with the 2^−∆∆Ct^ method.

### 2.5. Statistics Analysis

Data analysis of the gut microbiota differences among the groups was performed using Limma [44]. Data showed the differences between the control vs. DIO, control vs. DIO + MaR1, and DIO vs. DIO + MaR1 at the phylum, genus, and species level. The differences among groups were considered statistically significant when *p* < 0.05, and a log fold change (LogFC) between groups was shown to analyze upregulation and downregulation of the abundance of the bacterial profile. For *p* value adjustments, the false discovery rate (FDR) was applied, as described previously [45]. Further clustering analyses and graphical representations were performed using R/Bioconductor [43].

Comparisons among groups of the selected inflammatory genes were tested by one-way ANOVA followed by Bonferroni post hoc tests or with a two-tailed unpaired *t*-test and Mann–Whitney U test, once normality was calculated with a Kolmogorov–Smirnoff and Shapiro–Wilk test. Correlation analyses between phylum and inflammatory genes were determined by Pearson (parametric distribution) or Spearman’s tests (non-parametric distribution) and were considered significant when *p* < 0.05. Statistical analyses were performed using GraphPad Prism 8.0 (Graph-Pad Software INC, San Diego, CA, USA).

## 3. Results

### 3.1. Effects of Maresin 1 (MaR1) Treatment on the Expression of Inflammatory Genes in Colon Mucosa of Diet-Induced Obese (DIO) Mice

**Figure 1:**
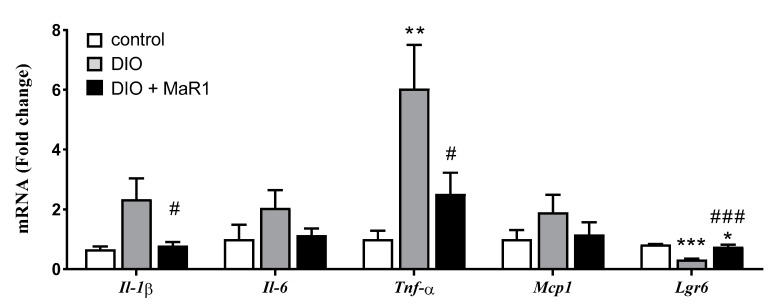
Effects of Maresin 1 (MaR1) on gene expression levels of *Il-1β*, *Il-6*, *Tnf-α*, *Mcp-1,* and *Lgr6* in colon. Diet-induced obese (DIO) mice were treated by oral gavage with MaR1 (50 μg/kg/day) for 10 days. Data are expressed as mean ± SE (*n* = 7–8). * *p* < 0.05, ** *p* < 0.01, *** *p* < 0.001 *vs.* control and ^#^ *p* < 0.05, ^###^ *p* < 0.001 vs. DIO mice.

To evaluate whether treatment with MaR1 could modulate the levels of proinflammatory cytokines, the expression levels of four representative inflammatory genes were measured. *Tnf-α* mRNA levels were clearly upregulated in the DIO group as compared with the control mice. Moreover, the expression of *Il-1β, Il-6,* and *Mcp-1* genes tended to be also elevated in the DIO mice as compared with the control animals (Figure 1). Significantly, treatment with MaR1 reversed the increase induced by the HFD on the expression of the proinflammatory cytokines *Il-1β* and *Tnf-α*. In addition, the MaR1-treated mice also exhibited moderate lower levels of the cytokine *Il-6* and the chemokine *Mcp-1*, although no significant differences were reached when compared to the DIO group (Figure 1).

Concerning the potential mechanisms involved in MaR1 actions, a recent study has discovered that LGR6 receptor activation mediated the pro-resolving functions of MaR1 in phagocytes [46]. The measurement of *Lgr6* mRNA levels in our mice model revealed that *Lgr6* levels were downregulated in the colon of the DIO mice, which was partially reversed by treatment with MaR1 (Figure 1), suggesting a potential involvement of LGR6 in the anti-inflammatory actions of MaR1 in the DIO mice.

### 3.2. Effects of MaR1 on Gut Microbiota Composition in DIO Mice

#### 3.2.1. Principal Component Analysis

**Figure 2:**
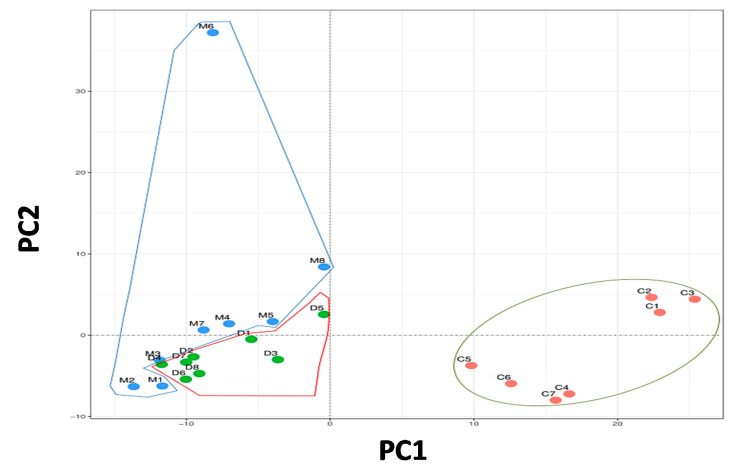
Principal component analysis (PCA) of gut microbiota metagenomes. The PCA analysis distribution of the faecal community respect to diet and treatment and the microbiota composition. (**C**) Control; (**D**) DIO; (**M**) DIO + MaR1. Groups were enclosed representing the similarity among the members of them. DIO mice were treated by oral gavage with MaR1 (50 μg/kg/day) for 10 days.

The composition of the bacterial community in the different experimental groups was assessed by the degree of bacterial taxonomic similarity using a metagenomics study. To compare community patterns, bacterial communities were grouped by a principal component analysis (PCA), which distinguished microbial communities, based both on diet and treatment (Figure 2). The plot was distributed as first principal component (PC1), explaining 30% of variation and second principal component (PC2), explaining a 25% of variation. The PC1 was mainly composed of Bacteroidetes, Cyanobacteres, Chlorobi, Proteobacteres and Verrucomicrobia, while the PC2 was mainly represented by Firmicutes, Deferribacteres, Synergistetes, Chloroflexi and Nitrospirae. The PCA showed a clear different bacterial distribution between the control group and both groups fed with the HFD (DIO and DIO + MaR1). However, the DIO individuals tended to be organized near PC1, while the DIO + MaR1 samples were distributed closer to PC2, a profile with a higher predominance of Firmicutes.

#### 3.2.2. Taxonomic Changes in Faecal Microbiome

##### Analysis of Taxonomic Changes at the Phylum Level

**Figure 3:**
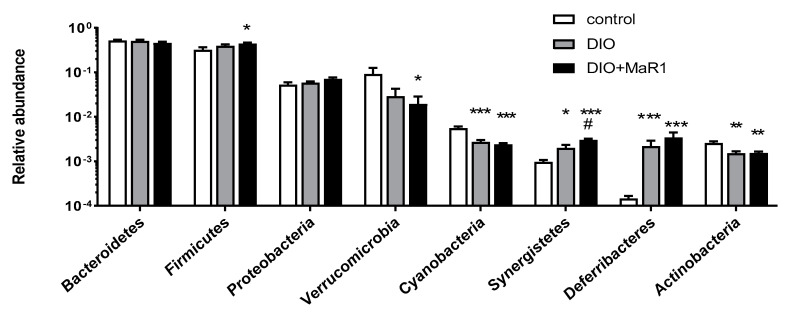
Relative abundance of major phyla in gut microbiota of the control and the DIO mice treated, or not treated, by oral gavage with MaR1 (50 μg/kg/day) for 10 days. Data are expressed as mean ± SE (*n* = 7–8). * *p* < 0.05, ** *p* < 0.01, *** *p* < 0.001 vs. control and # *p* < 0.05 vs. DIO.

To better characterize the changes in gut microbiota composition produced by the HFD and to determine if there was any effect of the treatment with MaR1, a comparison of the three experimental groups was performed at the phylum level. The metagenomics analysis identified 20 different phyla. The major phyla (representing more than the 99.5% of the total identified) are shown in Figure 3.

As previously described [47] the most abundant phyla were Bacteriodetes and Firmicutes. There were no significant changes detected between the control and DIO groups for both phyla, although the abundance of Firmicutes tended to be higher in the HFD-fed animals, and upregulated in the DIO + MaR1 group vs. the control group. Accordingly, the ratio of Firmicutes to Bacteroidetes, which was described to be increased in obesity [1,48] was higher in the groups fed with the HFD, reaching significant differences in the DIO mice treated with MaR1 as compared with the control mice (Figure S1).

In relation to the other phyla, the DIO mice did not exhibit any differences in Proteobacteria. However, the HFD induced a significant decrease in the content of Cyanobacteria and Actinobacteria as compared with the control group. Verrucomicrobia abundance was also reduced in the HFD-fed animals, but significant differences were only observed in the group treated with MaR1 (*p* < 0.05). In contrast, the abundance of Deferribacteres (*p* < 0.001) and Synergistetes was upregulated in both DIO groups, untreated and treated with MaR1, as compared with the control group (Figure 3). Remarkably, the relative abundance of Synergistetes significantly increased in DIO + MaR1 as compared to DIO, but this difference disappeared after adjustment by FDR (adj. *p* value = 0.056) [45].

Figure S2 represents the Heat map illustrating the relative abundance of the most different Phyla according to treatment group. Other less abundant phyla that were also significantly different between the control and the DIO mice and between the control and the DIO + MaR1 mice are described in Table S2. Thus, in both groups fed a HFD, a significant decrease was found for Tenericutes, Thermotogae, Spirochaetes, Caldithrix, and Chrysiogenetes. In contrast, the HFD-fed mice exhibited a higher content in Chloroflexi and Nitrospirae as compared with the control mice.

##### Analysis of Taxonomic Changes at the Genus Level

The analysis of taxonomic changes at the genus level showed that 115 of the 208 identified genera were differentially abundant in the DIO mice as compared with the control group (Table S3). Moreover, 26 genera were detected in different proportions between the DIO and the DIO + MaR1 groups (Table 1). Thus, 18 genera were decreased in the DIO + MaR1 mice, while eight were significantly increased (*p* < 0.05).

##### Analysis of Taxonomic Changes at the Species Level

At the species level, 143 of the 227 analyzed species revealed different richnesses in the DIO group as compared with the control mice. Interestingly, 12 of the 227 species exhibited different abundances between the DIO and the DIO + MaR1 groups. Indeed, seven species increased in the DIO + MaR1 group (*A. naturae, S. faecicanis, E. tangerine, P. fimeticola, D. litoralis, C. flavus,* and *P. xylanivorans*) (Table 2). In contrast, five species decreased in the DIO + MaR1 group as compared with DIO (*P. circumdentaria, M. enterobacteria, C. akajimensis, R. microfusus,* and *N. helminthoeca*) (Table 2).

**Figure 4:**
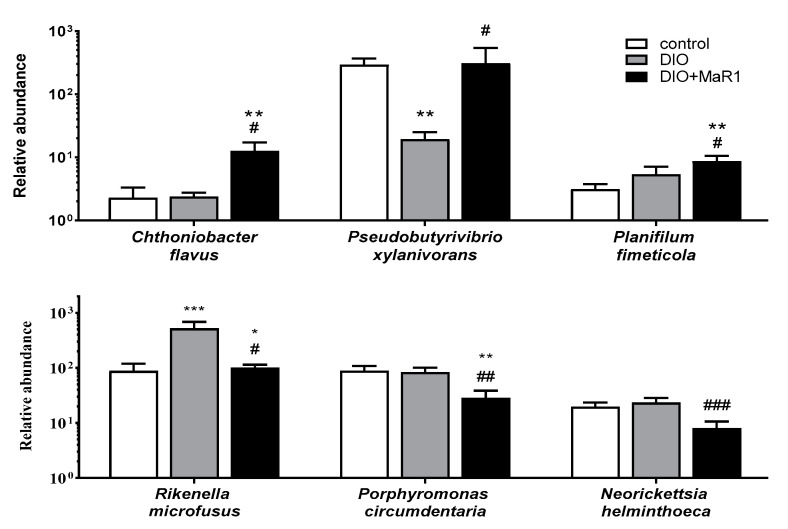
Relative abundance of species *C. flavus*, *P. xylanivorans*, *P. fimeticola R. microfusus*, *P. circumdentaria*, and *N. helminthoeca* in gut microbiota of the control and DIO mice treated, or not treated, by oral gavage with MaR1 (50 μg/kg/day) for 10 days. Data are expressed as mean ± SE (*n* = 7–8). * *p* < 0.05, ** *p* < 0.01, *** *p* < 0.001 vs. control and # *p* < 0.05, ## *p* < 0.01, ### *p* < 0.001 vs. DIO.

The abundance of some of these species is shown in Figure 4. Interestingly, the HFD induced a significant decrease in the abundance of *P. xylanivorans*, which was completely reversed by the treatment with MaR1, reaching similar levels to those obtained for the control group. In addition, treatment with MaR1 induced an increase in the *C. flavus* and *P. fimeticola* levels (Figure 4). In contrast, MaR1 administration decreased the content of *P. circumdentaria,* and *N. helminthoeca* levels. More interestingly, the DIO group exhibited a significant increase in *R. microfusus* abundance, which was reversed in those obese animals treated with MaR1 (Figure 4).

### 3.3. Correlation between Specific Inflammatory Genes in Colonic Mucosa and the Gut Microbiota Composition at the Phylum Level

A correlation analysis was performed to test the potential associations between the expression of proinflammatory genes in the colonic mucosa and gut microbiota. Our data revealed a robust positive association between the phylum Deferribacteres with *Il-1β* mRNA expression in the colon (*r* = 0.514 and *p* = 0.03). There were no other significant correlations found between other inflammatory genes and gut microbiota phyla. However, the expression of *Tnf-α* showed a tendency to correlate positively with Deferribacteres (*r* = 0.380 and *p* = 0.081) and negatively with Spirochaetes (*r* = −0.379 and *p* = 0.081).

## 4. Discussion

The present study shows that DIO mice exhibited an upregulation of colon proinflammatory biomarkers expression such as *Tnf-α* and a trend to increase *Mcp-1*, *Il-6*, and *Il-1β*. These results are in accordance with previous reports that described an enhanced gene expression of *Tnf-α* and *Il-1β* during the progression of intestinal inflammation in HFD-induced obese mice [49,50]. Thus, these studies and our data suggest an increased local production of inflammatory cytokines in the bowel of mice after receiving the HFD.

Bento et al. [22] reported that treatments with some SPMs (RvD1, RvD2, and 17R-HDHA) were effective in preventing gut inflammation in experimental mice models of colitis, reducing colonic cytokine levels of *TNF-α*, *IL-1β*, and other inflammatory markers. Related to this first study, Warner et al. [51] found that both the increased levels of endogenous n-3 PUFAs, which modified the ratio n-6/n-3 PUFAs, and the RvD1 treatment ameliorated intestinal inflammation in mice, in this case mediated by ethanol. RvD1 reduced the neutrophil infiltration induced by ethanol + LPS. Moreover, the expression of *Il-6*, *Cxcl1*, and *Tnf-α* were reduced through RvD1 treatment [51]. In a similar way, MaR1 was also effective in reducing different stages of gut inflammation in colitic mice, downregulating some proinflammatory mediators such as *IL-1β*, *TNF-α*, *IL-6*, and *INF-γ* [31]. Moreover, a recent study by our group described that in DIO mice, oral gavage of MaR1 reversed the upregulation of proinflammatory cytokines found in jejunal mucosa [33].

The current study shows that mice treated with MaR1, also exhibited anti-inflammatory actions in colonic mucosa, as it reduced mRNA levels of proinflammatory *Il-1β* and *Tnf-α* in DIO mice. An assessment of proinflammatory cytokines at the protein level would have been of interest to determine whether changes at the protein level would mimic those observed at the mRNA level. However, because of the limitation in the available colon samples, we only assessed mRNA expression of proinflammatory genes, considering that in the study by Wilk et al. [52] with colitic mice the results between protein levels and mRNA expression levels in large intestine were correlated. On the other hand, MaR1 seems to be effective in resolving both the experimentally induced acute and chronic colitis described by Marcon et al. [31] and the low-grade chronic inflammation induced by the HFD in the colon of DIO mice. Ding et al. [8] provided evidences that intestinal inflammation was an early consequence of the HFD, which could contribute to obesity-associated insulin resistance. We have previously reported that MaR1-treated mice, showed a better glycaemic profile with a reduction in fasting glucose and fasting insulin [21], and it could be suggested that the reduction of colonic mucosa inflammation, induced by MaR1, could also be potentially contributing to the insulin-sensitizing effects observed for this DHA-derived SPM. In this context, interactions among a HFD, the release of proinflammatory cytokines in small intestine, and gut microbiota have been suggested, which could affect whole-body metabolism [8].

Several reports have strongly suggested that gut microbiota could play an important role in the pathogenesis of obesity, inflammatory bowel, and type 2 diabetes [53,54]. In the present study, we also aimed to understand changes in gut microbiota composition in response to a HFD and MaR1 treatment and its potential association with the intestinal inflammation observed in DIO mice. To our knowledge, this is the first study evaluating the effects of a SPM by oral gavage administration in gut microbiota.

Current metagenomic data have revealed important differences in bacterial communities in response to different diets (HFD vs. control diet) which is in agreement to what has been previously described in other studies [50,55,56]. According to other reports, Firmicutes and Bacteroidetes were the major phyla represented in our study [1,47]. In this context, several studies have described that a HFD led to an increase in the Firmicutes/Bacteroidetes ratio in gut microbiome [1,53,55,56]. In our findings, the DIO group showed a marginal upregulation of the Firmicutes/Bacteroidetes ratio. Similarly, other publications did not find significant associations between the HFD-fed mice and the downregulation of Bacteroidetes and upregulation of Firmicutes [57,58]. Regarding the effects of MaR1 administration on the gut microbiota of DIO mice, the PCA analysis disclosed that both groups fed a HFD had a substantial similarity in bacterial taxonomic. However, the group treated with MaR1 exhibited a significant increase of Firmicutes and in the Firmicutes/Bacteroidetes ratio. These results could suggest that MaR1 could potentiate the changes in Firmicutes commonly associated with a HFD. These data contrast with previous findings that have reported an increase in Bacteroidetes abundance after treatment with RvD1 (5 μg/kg, i.p. 16 days) and fish oil supplementation [59]. Moreover, treatments to overcome obesity such as dietary intervention with either fat- or carbohydrate-restricted diets or bariatric surgery seems to reverse this ratio, by increasing Bacteroidetes levels to a microbiota profile more similar to that observed in lean individuals [1]. However, the relationships between Bacteroidetes, Firmicutes, and metabolic disorders such as type 2 diabetes and NAFLD are unclear. For instance, several trials have revealed a higher relative abundance of Bacteroidetes, and lower population of Firmicutes in subjects/animal models suffering from type 2 diabetes or NASH/NAFLD as compared with control mice [60,61]. Furthermore, treatment with Flos Lonicera combined with Metformin tended to reduce the relative abundance of Bacteroidetes, while increasing Firmicutes levels in Otsuka Long-Evans Tokushima Fatty (OLETF) rats (a model of genetic type 2 diabetes and NAFLD) in parallel with an improvement in hepatosteatosis and glucose intolerance [61]. In obese mice, MaR1 also alleviates liver steatosis and improves insulin sensitivity [21,26]. In relation to other phyla, our study showed that Proteobacteria remained without apparent changes after HFD feeding. Walker et al. [62] revealed a different abundance of Proteobacteria in response to feeding between two different C57BL/6 strains with different susceptibility to diet-induced obesity. The abundance of Verrucomicrobia was not significantly changed in untreated HFD-fed mice, which was in accordance with another study [56]. Other trials described a negative correlation between Verrucomicrobia, and more specifically *A. muciniphila*, with body weight [63].

In the present investigation, other quite abundant phyla were Deferribacteres and Synergistetes, which showed important differences among the mice groups. In the HFD-induced obese mice, the abundance of Deferribacteres and Synergistetes increased significantly in contrast to the control group, which was in accordance with previous publications [56,62]. The abundance of Deferribacteres has been linked with exacerbated intestinal inflammation in previous studies of colitis in mice [64]. In this way, our study has revealed that the gene expression of the proinflammatory cytokine *Il-1β* in colonic mucosa was positively correlated with the upregulation of Deferribacteres. Nevertheless, treatment with MaR1 caused a significant reduction of *Il-1β* cytokine expression, although it was unable to reduce the Deferribacteres levels induced by the HFD. This observation suggests that the pro-resolutive effect of MaR1 in colon inflammation is not related to changes in Deferribacteres abundance, indicating that this effect could be linked to different mechanisms. Synergistetes have been poorly described in gut microbiota of mice. A recent study observed no changes in Synergistetes in gut microbiota of obese women with or without metabolic syndrome vs. lean [65]. Our current study found that treatment with MaR1 did not reverse the upregulation of Synergistetes observed in DIO mice. Further studies should be done to better characterize the physiological specific role of Synergistetes in gut microbiota and their potential impact on health, as well as to evaluate whether a longer treatment with MaR1 could modulate the changes induced by the HFD.

The genus *Rikenella* has been determined to be a common member in the digestive tract and a HFD tended to reduce *R. microfusus* levels, but this effect was counteracted after an infusion treatment with green tea [66]. In contrast, our study showed that *R. microfusus* was upregulated in response to HFD feeding, while MaR1 treatment reversed the abundance, reaching similar levels of abundance to that of the control group.

The MaR1-treated mice showed a downregulation in *P. circumdentaria* and *N. helminthoeca* in contrast to the DIO untreated mice. *P. circumdentaria* has been reported in the oral cavity of cats, and related with periodontal diseases [67]. *N. helminthoeca* has been associated with a pathogenic profile, parasitizing nematodes [68]. Therefore, the reduction of these apparently pathogenic species could be positive for the gut microbiota community. *Pseudobutyrivibrio xylanivorans* was upregulated after MaR1 treatment in contrast to DIO mice, reaching similar levels of abundance to that of the control group. In the study by Cepeljnik et al. [69], *P. xylanivorans* was isolated from rumen, and was related to a higher production of butyrate and bacteriocin, suggesting that these characteristics could favour its use as probiotic bacteria [69]. Bacterocin production works for controlling pathogenic bacteria, including some strains of rumen bacteria, and *Salmonella* and *Escherichia coli* [69]. Furthermore, butyrate production has been associated with a protective role against colon cancer, colitis, and to display anti-inflammatory effects [70]. Thus, *P. xylanivorans* was enhanced with the MaR1 treatment, and it is a butyrate-producer bacterium. This is an interesting finding of our study, which could suggest that the raising of this species could contribute to the anti-inflammatory effect in the colonic mucosa caused by the HFD. Moreover, *P. xylanivorans* has the ability to transform linoleic acid into conjugated linoleic acid and this metabolite is a potential inducer of apoptosis. Thereby, these bacteria could be considered to be a potential probiotic with anticancer activity [71]. Nevertheless, more experiments are needed to better outline the potential benefits of *P. xylanivorans* and to determine its potential impact on obesity and inflammatory processes.

A limitation of the current study is that the gut microbiota analyses were performed at the end of the treatment. It would be of interest to perform studies comparing the microbiota composition of each animal before and after the treatment. In this context, the current data suggest that a longer treatment with MaR1 could be necessary to observe more relevant changes on gut microbiota composition.

An important question is the characterization of the pathway or pathways through which MaR1 ameliorates the gut inflammation and affect the population of bacteria. Two recent studies have discovered that two types of receptor molecules could be mediating the pro-resolving properties of MaR1, i.e., the retinoic acid-related orphan receptor α (RORα) and LGR6 [30,46,72]. Interestingly, RORα seems to be crucial for attenuated inflammatory response to maintain intestinal homeostasis [73], and therefore we can speculate that RORα activation could be involved in MaR1 beneficial actions on obesity-induced colon inflammation. LGR6 is a very quick acting and highly specific receptor for MaR1-induced phagocytosis and efferocytosis in human macrophages [46]. Interestingly, our study revealed that the expression of *Lgr6* is markedly inhibited in the colon of obese mice in parallel with the stimulation of the expression of proinflammatory cytokines. MaR1 administration partially reverses the inhibitory effects of obesity on colonic *Lgr6* levels, suggesting the potential involvement of LGR6 in the anti-inflammatory properties of MaR1 in the inflamed colon of obese mice. Further studies are needed to better characterize the role of these receptors and the signalling pathways involved in MaR1 actions in intestinal resolution of chronic inflammation and gut microbiota dysbiosis associated with obesity.

## 5. Conclusions

To sum up, our study revealed that MaR1 administration can ameliorate the chronic low-grade inflammatory state in colonic mucosa related to obesity and partially compensate changes in gut microbiota as a consequence of a HFD. However, more studies are required to completely clarify the role and therapeutic potential of MaR1 and other SPMs in obesity-induced intestinal inflammation and gut dysbiosis.

## Figures and Tables

**Table 1:** Differential bacterial taxonomic profile at the genus level in the DIO + MaR1 vs. the DIO mice.

Downregulated in DIO + MaR1 vs. DIO			Upregulated in DIO + MaR1 vs. DIO		
*Genus*	LogFC	*p*	*Genus*	LogFC	*P*
*Gluconacetobacter*	−2.007	0.001	*Pseudobutyrivibrio*	2.771	0.009
*Pseudomonas*	−1.595	0.001	*Chthoniobacter*	1.723	0.010
*Psychrobacter*	−0.841	0.001	*Planifilum*	1.190	0.022
*Hymenobacter*	−0.776	0.002	*Sphaerisporangium*	1.146	0.026
*Microvirus*	−1.356	0.004	*Dethiosulfovibrio*	1.031	0.033
*Neorickettsia*	−1.068	0.007	*Euzebya*	0.879	0.044
*Rikenella*	−1.288	0.011	*Actinomyces*	0.681	0.045
*Thiothrix*	−0.584	0.011	*Faecalibacterium*	1.000	0.049
*Candidatus* Amoebophilus	−0.758	0.012			
*Emticicia*	−0.899	0.023			
*Gillisia*	−0.670	0.032			
*Tetragenococcus*	−0.549	0.032			
*Thiothrix*	−0.584	0.011			
*Enterococcus*	−1.378	0.033			
*Polaribacter*	−0.792	0.037			
*Paraprevotella*	−0.933	0.039			
*Candidatus* Glomeribacter	−0.647	0.042			
*Coraliomargarita*	−1.223	0.049			

Negative log fold change (LogFC) was related to lower representation in the DIO + MaR1 as compared with the DIO, whereas positive LogFC corresponds to those genera that increased in the DIO + MaR1 as compared with the DIO. Genera were organized by their significance (*p*). DIO mice were treated by oral gavage with Maresin 1 (MaR1, 50 μg/kg/day) for 10 days.

**Table 2:** Differential bacterial taxonomic profile at the species level in the DIO + MaR1 vs. DIO mice.

Downregulated in DIO + MaR1 vs. DIO			Upregulated in DIO + MaR1 vs. DIO		
*Species*	LogFC	*p*	*Species*	LogFC	*p*
*Porphyromonas circumdentaria*	−1.055	0.003	*Actinomyces naturae*	0.853	0.019
*Microvirus enterobacteria*	−1.264	0.007	*Slackia faecicanis*	0.959	0.025
*Coraliomargarita akajimensis*	−1.201	0.011	*Euzebya* *tangerine*	1.051	0.027
*Rikenella microfusus*	−1.116	0.015	*Planifilum fimeticola*	1.349	0.031
*Neorickettsia helminthoeca*	−0.928	0.016	*Desulfovibrio litoralis*	1.397	0.039
			*Chthoniobacter flavus*	1.742	0.041
			*Pseudobutyrivibrio xylanivorans*	2.956	0.045

Negative log fold change (LogFC) was related to lower representation in DIO + MaR1 as compared with DIO, whereas positive LogFC corresponds to those species that increased in DIO + MaR1 as compared with the DIO mice. Species were organized by their significance (*p*). DIO mice were treated by oral gavage with MaR1 (50 μg/kg/day) for 10 days.

## Supplementary Materials

The following data are available online at https://www.mdpi.com/2076-2607/8/8/1156/s1, Figure S1: Firmicutes/Bacteroidetes ratio, Figure S2: Heat map illustrating the relative abundance of the most different phyla according to treatment, Table S1: TaqMan assays used for the gene expression analyses, Table S2: Differential bacterial taxonomic profile at the phylum level in DIO vs. control and DIO + MaR1 vs. control mice, Table S3: Differential bacterial taxonomic profile at the genus level in DIO vs. control mice.
