# Supplementary material for: Effects of Maresin 1 (MaR1) on Colonic Inflammation and Gut Dysbiosis in Diet-Induced Obese Mice
Source: Microorganisms. 2020 Jul 30;8(8):1156. doi: 10.3390/microorganisms8081156 (PMC7465372; doi:10.3390/microorganisms8081156)
Supplement: Supplementary file 1 [file microorganisms-08-01156-s001.pdf]

Supplementary Materials:

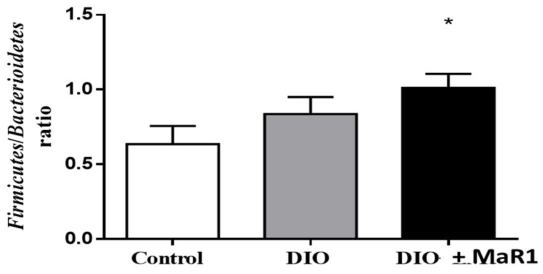

Figure S1. *Firmicutes/Bacteroidetes* ratio in Control and DIO mice treated by oral gavage with Maresin 1 (MaR1; 50 μg/kg/day) or treated with vehicle for 10 days. Data are expressed as mean ± SE (n=7-8). (□) Control; (■) DIO; (■) DIO+ MaR1. *p<0.05 *vs.* Control.

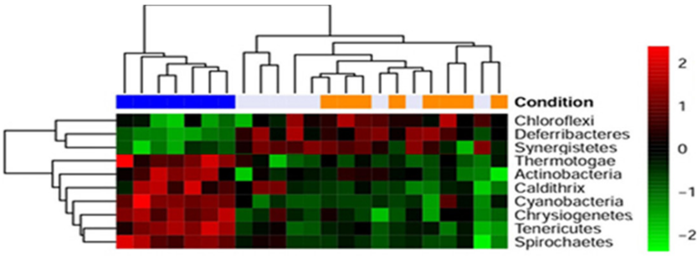

Figure S2. Heat map illustrating the relative abundance of the most different *Phyla* according to treatment group. (■) Control; (■) DIO; (■) DIO+MaR1. In the heatmap, red and green indicate high and low abundance respectively relative to the mean abundance of all samples for each *Phyla*. DIO mice were treated by oral gavage with Maresin 1 (MaR1; 50 μg/kg/day) for 10 days.

Table S1. Taqman assays used for the gene expression analyses.

**Name**	**Gen**	**TaqMan probe**
**Interleukin 6**	*Il-6*	Mm00446190_m1
**Interleukin 1 beta**	*Il-1β*	Mm00434228_ml
**Monocyte chemoattractant protein 1**	*Mcp-1*	Mm00441242_ml
**Tumor Necrosis Factor alpha**	*Tnf-α*	Mm00443258_ml
**18S ribosomal RNA**	*18S*	Mm03928990_g1

Table S2. Differential bacterial taxonomic profile at the *Phylum* level in DIO *vs*. Control and DIO+MaR1 *vs*. Control mice.

**Down-regulated DIO vs. Control**				**Down-regulated DIO+MaR1 *vs*. Control**		
Phylum	**LogFC**	***p***	**adj. *p* Value**	**LogFC**	***p***	**adj. *p* Value**
Tenericutes	-3.316	<0.001	<0.001	-3.542	<0.001	<0.001
Caldithrix	-2.358	0.001	0.003	-1.894	0.005	0.011
Spirochaetes	-2.217	<0.001	<0.001	-2.160	<0.001	<0.001
Verrucomicrobia	-1.862	0.050	0.087	-2.468	0.011	0.022
Chrysiogenetes	-1.470	<0.001	<0.001	-1.539	<0.001	<0.001
Thermotogae	-1.328	<0.001	<0.001	-1.610	<0.001	<0.001
Cyanobacteria	-1.039	<0.001	<0.001	-1.186	<0.001	<0.001
Actinobacteria	-0.770	0.002	0.005	-0.743	0.003	0.007

**Up-regulated DIO *vs*. Control**				**Up-regulated DIO+ MaR1 *vs*. Control**		
Phylum	**LogFC**	***p***	**adj. *p* Value**	**LogFC**	***p***	**adj. *p* Value**
Proteobacteria	0.225	0.334	0.467	0.480	0.046	0.065
Firmicutes	0.385	0.106	0.171	0.556	0.023	0.037
Chloroflexi	0.605	0.003	0.007	0.442	0.025	0.037
Nitrospirae	0.671	0.040	0.077	0.745	0.024	0.037
Synergistetes	0.935	0.005	0.011	1.667	<0.001	<0.001
Deferribacteres	3.508	<0.001	<0.001	3.809	<0.001	<0.001

Negative Log Fold Change (LogFC) was related to lower representation in DIO or DIO+MaR1 compared to Control group, whereas positive LogFC corresponds to those *Phyla* that were increased in DIO or DIO+MaR1 compared with Control mice. *Phyla* were organized by their LogFC. DIO mice were treated by oral gavage with Maresin 1 (MaR1; 50 μg/kg/day) for 10 days.

Table S3. Differential bacterial taxonomic profile at the *Genus* level in DIO *vs*. Control mice.

**Down-regulated in DIO *vs.* Control**			**Up-regulated in DIO *vs.* Control**		
Genus	**LogFC**	***p***	***Genus***	**LogFC**	***p***
Anaeroplasma	-7.855	6.99×10^{-11}	*Caldilinea*	0.484	0.043
Sutterella	-7.764	2.98×10^{-09}	*Moraxella*	0.641	0.033
Johnsonella	-7.137	3.74×10^{-17}	*Deferribacter*	0.724	0.023
Coprococcus	-6.327	7.67×10^{-06}	*Methylobacterium*	0.802	0.029
Allobaculum	-6.151	2.27×10^{-06}	*Ehrlichia*	0.819	0.032
Anaerostipes	-6.067	6.59×10^{-11}	*Deinococcus*	0.836	0.026
Limnobacter	-5.800	3.63×10^{-08}	*Microcoleus*	0.854	0.010
Desulfosarcina	-5.609	2.62×10^{-08}	*Amoebophilus*	0.889	0.005
Brachyspira	-5.035	2.03×10^{-11}	*Paenibacillus*	0.900	0.002
Bifidobacterium	-4.636	2.78×10^{-09}	*Streptomyces*	0.904	0.006
Turicibacter	-4.396	6.05×10^{-04}	*Adlercreutzia*	0.955	0.017
Geobacter	-4.185	1.26×10^{-05}	*Dehalobacterium*	0.969	0.029
Roseburia	-3.873	5.55×10^{-04}	*Streptococcus*	1.085	0.001
Novispirillum	-3.805	3.41×10^{-03}	*Oscillospira*	1.162	0.005
Sarcina	-3.800	1.15×10^{-02}	*Pseudomonas*	1.183	0.013
Pseudobutyrivibrio	-3.653	1.31×10^{-03}	*Gluconobacter*	1.260	0.038
Anaerovibrio	-3.620	2.35×10^{-08}	*Enterobacter*	1.429	0.035
Lachnobacterium	-3.512	1.59×10^{-05}	*Flavobacterium*	1.436	0.003
Dysgonomonas	-3.418	3.94×10^{-11}	*Helicobacter*	1.460	<0.001
Prosthecobacter	-3.377	2.45×10^{-02}	*Desulfovibrio*	1.476	0.001
Acholeplasma	-3.171	1.25×10^{-04}	*Slackia*	1.487	0.031
Mesoplasma	-3.134	2.69×10^{-04}	*Kitasatospora*	1.496	0.016
Luteolibacter	-3.076	4.24×10^{-02}	*Pedobacter*	1.502	<0.001
Legionella	-3.049	2.62×10^{-09}	*Chromatium*	1.520	0.002
Uliginosibacterium	-2.716	9.35×10^{-06}	*Alkaliphilus*	1.717	0.000
Eggerthella	-2.661	7.98×10^{-05}	*Thermobaculum*	1.770	0.003
Eubacterium	-2.638	6.29×10^{-05}	*Holdemania*	1.787	0.005

Table S3 continuation

**Down-regulated in DIO *vs.* Control**		
Genus	**LogFC**	***p***
Lysobacter	-2.603	$2.01 x10^{02}$
Caldithrix	-2.444	$6.28 x10^{04}$
Pectinatus	-2.435	$3.28x 10^{05}$
Marinitoga	-2.371	$4.30 x10^{07}$
Desulfotomaculum	-2.334	$2.83 x10^{04}$
Prevotella	-2.331	$1.17 x10^{02}$
Faecalibacterium	-2.210	$1.79 x10^{04}$
Parapedobacter	-2.135	$2.13 x10^{05}$
Mannheimia	-2.119	$5.64 x10^{04}$
Petrotoga	-2.039	$1.24 x10^{03}$
Rhodospirillum	-2.023	$2.48 x10^{02}$
Sedimentibacter	-1.932	$7.17 x10^{06}$
Heliorestis	-1.803	$2.49 x10^{05}$
Muricauda	-1.792	$1.64 x10^{06}$
Selenomonas	-1.762	$5.57 x10^{04}$
Acinetobacter	-1.565	$1.92 x10^{03}$
Desulfurispirillum	-1.556	$1.63 x10^{05}$
Coraliomargarita	-1.516	$2.01 x10^{02}$
Microbacterium	-1.400	$1.94 x10^{03}$
Butyrivibrio	-1.357	$4.22 x10^{02}$
Stenotrophomonas	-1.282	$1.77 x10^{02}$
Candidatus Phytoplasma	-1.249	$8.32 x10^{04}$
Butyricimonas	-1.229	$5.14 x10^{03}$
Emticicia	-1.182	$5.08 x10^{03}$
Caldicellulosiruptor	-1.169	$3.32 x10^{03}$
Luteibacter	-1.165	$2.26 x10^{02}$
Kribbella	-1.159	$2.39 x10^{02}$
Bradyrhizobium	-1.151	$1.09 x10^{03}$
Parabacteroides	-1.125	$3.97 x10^{03}$
Paraprevotella	-0.982	$3.58 x10^{02}$
Dolichospermum	-0.978	$2.33 x10^{02}$
Olivibacter	-0.975	$6.84 x10^{04}$
Marinobacter	-0.917	$2.95 x10^{03}$
Dyadobacter	-0.877	$8.02 x10^{03}$
Fervidobacterium	-0.796	$2.15 x10^{02}$
Rhodobacter	-0.786	$3.47 x10^{02}$
Thermus	-0.742	$7.25 x10^{03}$

**Up-regulated in DIO *vs.* Control**		
Genus	**LogFC**	***p***
Ruminococcus	1.807	<0.001
Capnocytophaga	1.860	<0.001
Segetibacter	2.018	<0.001
Rhodothermus	2.051	<0.001
Escherichia	2.068	0.029
Caldanaerobacter	2.069	<0.001
Smithella	2.196	<0.001
Sphaerisporangium	2.218	<0.001
Corynebacterium	2.274	<0.001
Gluconacetobacter	2.283	<0.001
Lachnospira	2.303	0.001
Rikenella	2.325	<0.001
Odoribacter	2.380	<0.001
Gillisia	2.478	<0.001
Caloramator	2.616	<0.001
Vagococcus	3.060	<0.001
Acidaminobacter	3.127	<0.001
Peptococcus	3.289	<0.001
Enterococcus	3.727	<0.001
Bilophila	3.758	<0.001
Mucispirillum	3.793	<0.001
Aquimarina	4.552	<0.001
Anaerotruncus	4.761	<0.001
Lactococcus	8.302	<0.001

Negative Log Fold Change (LogFC) was related to lower representation in DIO compared to Control whereas Positive LogFC corresponds to those *Genera* that were increased in DIO compared with Control. *Genera* were organized by their LogFC.
